# The Evolving View of Uremic Toxicity

**DOI:** 10.3390/toxins14040274

**Published:** 2022-04-12

**Authors:** Bjorn Meijers, Jerome Lowenstein

**Affiliations:** 1 Nephrology and Transplantation Unit, University Hospitals Leuven, 30000 Leuven, Belgium; bjorn.meijers@uzleuven.be; 2 Laboratory of Nephrology, Katholieke Universiteit Leuven, 30000 Leuven, Belgium; 3 Nephrology Division, NYU Langone Medical Center, New York, NY 10016, USA

**Keywords:** protein-bound solute, uremia, organic anion transporter

## Abstract

Indoxyl sulfate, closely related to indigo, a dye valued for it binding to cloth, has been recognized as a protein-bound solute bound to albumin, present in increased concentration in the serum of patients with impaired glomerular filtration (13). The early studies of Niwa identified indoxyl sulfate as a toxin capable of accelerating the rate of renal damage in subtotal nephrectomized rats (18). Over the past decade other protein-bound solutes have been identified in the plasma of patients with impaired glomerular filtration. Although the early studies, focused on the kidney, identified indoxyl sulfate as a toxic waste product dependent on the kidney for its removal, subsequent observations have identified organic anion transporters on many non-renal tissue, leading to the view that indoxyl sulfate is part of a systemic signaling system.

## 1. The Concept of Uremic Toxicity

Urine has been linked to human disease since ancient times. Exploration of its characteristics by means of the senses is arguably the oldest medical diagnostic test and marks the beginning of laboratory medicine [1]. Uroscopy became one of the prime tools for many generations of physicians [2]. Although this art has been practiced for more than 5000 years, it was not until the end of the 17th century that scientists realized that urine contained solutes. Herman Boerhave described a method to purify the ‘sal nativus urinae’ (the native salt of the urine) in his magnum opus “elementa chemicae”, published in Paris in 1727–1732 [3]. This salt later became known as urea, an end-product of protein metabolism in man. The meaning of this observation was not immediately clear, and only in the 19th century it was confirmed that urea, present in the blood, is excreted by the kidneys [4].

At around the same time, the London-based physician, Richard Bright, provided the hallmark characteristics of a syndrome that soon became known as Bright’s disease. He described a number of individuals with ‘dropsy’ (swelling due to edema), an increased bleeding tendency, vision disturbances, convulsions and coma ultimately leading to the death of those affected. He noted that the urine in these individuals contained red blood cells and, most often, significant amounts of protein [5].

Friedrich Theodor von Frerichs, a German physician, studied a number of patients with Bright’s disease and described three stages of the disease in “Die Brightische Nierenkrankheit und deren Behandlung” [6]. He combined the understanding of organic chemistry with the insights in human disease and in 1851 coined the term ‘uremic intoxication’. This led to a quest to find a way to reverse the accumulation of these molecules, though Frerichs himself expressed the opinion that urea accumulation itself probably did not cause the symptoms of uremia [6].

## 2. The Impact of Hemodialysis on the Concept of Uremic Toxicity

A hallmark paper by John Jacob Abel and colleagues reported the application of a device that permitted dialysis of blood in experimental animals [7]. They were able to remove substantial amounts of urea and other amino acid conjugates from the blood and performed basic chemical analyses to characterize some of these components [7]. In the same year, the New York Times described how blood could circulate through this machine, how coagulation could be overcome by using the anticoagulant, hirudoid, and reported that many substances could be eliminated from the blood [8]. Despite the wide acclaim, his device probably was not conceived as a therapy for uremic intoxication, and there is no written evidence of any clinical application of this apparatus in patients with end-stage kidney disease.

The German physician Georg Haas is credited for performing the first, albeit unsuccessful, dialysis to treat patients with end-stage kidney disease [9]. His attempt was more to show the feasibility of extracorporeal blood circulation than an actual treatment and only lasted about fifteen minutes [10]. Several years later, with longer treatments, he was able to detect several solutes in the dialysate bath including creatinine, phenol and indican, the latter now better known as indoxyl sulphate [11]. Despite these efforts, he was unable to show the clinical benefit of these treatments.

Willem Kolff, a Dutch physician working in a small hospital in the Netherlands, was well aware that urea and creatinine concentrations were elevated in patients who presented with the clinical findings designated as “uremia”, and that some of these “toxins” were small enough to pass across a semi-permeable membrane. Although aware of previous failures, Kolff proceeded to design a dialyzer that he used to treat patients with uremia [12,13]. Based upon initial data, he calculated how long a treatment would be required to remove sufficient urea to return blood concentrations to substantially lower levels. His main research work took place during World War II, and he was forced to improvise to a great extent under the occupying forces. Kolff’s initial work on dialysis was almost a junk-yard challenge and, as the story goes, he built his machine from washing machine parts, parts of a disabled warplane and sausage skins. He reported notable improvement following a single dialysis treatment. Although the first thirteen patients died soon after their dialysis, this was seen as a “proof of principle”; the fourteenth patient survived and the era of hemodialysis for kidney failure began.

Repeated hemodialysis was initially limited to patients who were being prepared for kidney transplant. Subsequently, repeated hemodialysis treatments for chronic renal failure followed the work of Belding Scribner who developed the arteriovenous shunt and dialysis cartridge to allow for repeated (maintenance) hemodialysis treatment. While thrice or biweekly hemodialysis came to be widely practiced, initially, little attention was paid to the nature of the toxins removed. It was recognized that solutes which were readily measured, such as urea and creatinine, were not toxic. They were assumed to be surrogates for uremic toxins that were removed by hemodialysis. Scribner and his coworkers reported [14]—and subsequently many studies have confirmed [15]—that maintenance hemodialysis, while effective in controlling uremic symptoms and maintaining fluid balance, was attended by a 50% three year mortality rate among patients undergoing maintenance hemodialysis. Notably, this late mortality was largely cardiovascular heart failure, arrhythmia and sudden death. Changes in modes of hemodialysis, frequency, membrane composition and other changes did not materially reduce this excess mortality [16,17].

## 3. Indoxyl Sulphate Revisited

A significant finding by Niwa and his associates in 1994 opened a new era in the understanding of uremic toxins [18,19,20]. They observed that the administration of a small molecule, indoxyl sulfate, as well as its metabolic precursor, indole, accelerated the development of uremia in partially nephrectomized rats. This greatly stimulated interest in uremic toxins and was soon followed by the establishment of the Eutox consortium [21,22,23,24]. Reviewing the published literature and confirming many of the reports, these investigators identified more than 100 solutes reported to be present at increased concentrations in the serum of patients with renal disease. Roughly 50% were small water-soluble readily filtered solutes such as urea and creatinine, and 25% were well-recognized macromolecules, e.g., microglobulins and complement, known to be too large to be filtered across the glomerular membrane. Attention was focused on a group of small solutes whose binding to protein (usually albumin) rendered them too large to be filtered across the glomerular membrane [25]. It was soon recognized that many of these protein-bound solutes were actively transported from the plasma to the tubular lumen by organic anion or cation transporters (OATs or OCTs) in the proximal renal tubule [26,27,28,29,30,31,32,33]. Subsequent studies have recognized that several protein-bound solutes, notably indoxyl sulfate, P-cresyl sulfate and kynurenine, all poorly dialyzable, accumulate as nephron function declines and might represent uremic toxins [34,35,36,37,38].

In a seminal study, Jhawar and coworkers examined the effects of indoxyl sulfate on gene expression in cultured human renal tubular cells incubated with normal (control) or uremic plasma (pre- and post-hemodialysis) [39]. After a 24 h incubation, RNA was harvested and analysis by gene chip identified 2000 genes that were “dysregulated”, defined as a 10% increase or decrease in gene expression.

**Figure 1:**
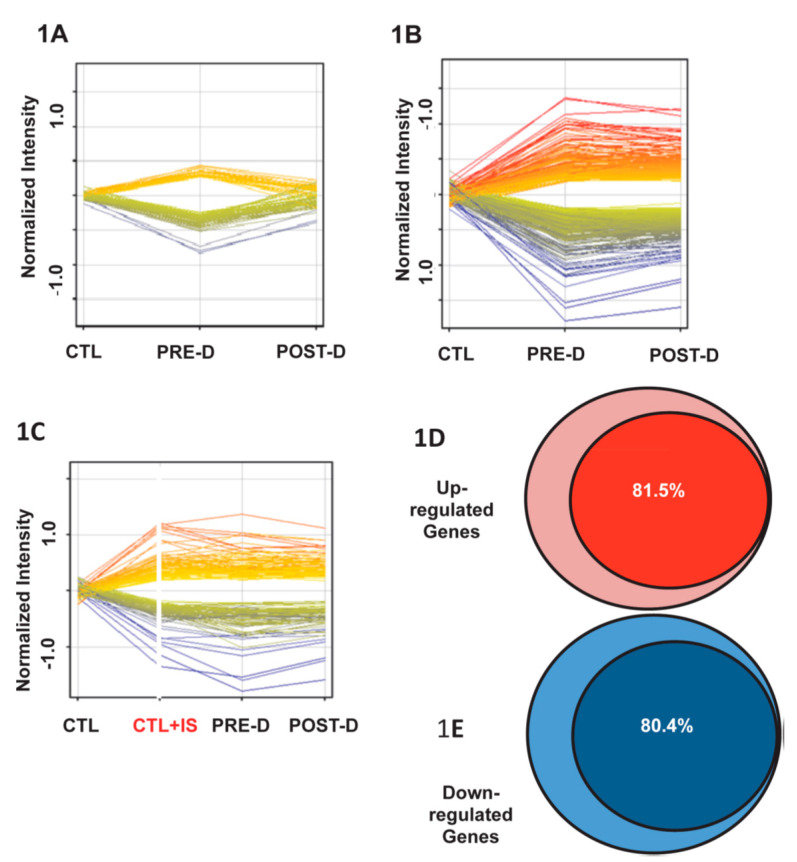
From Jhawar S et al. PLoS ONE 10(3): e0118703.Figure 1. Gene expression in reporter renal tubular cells. (**A**): Genes that returned to baseline post-dialysis. Each individual line represents the average value in the 10 uremic subjects for each gene that was dysregulated. A total of 282 genes were upregulated (displayed as yellow to red lines) and 255 genes were downregulated (displayed as green to blue lines) in the pre-dialysis samples as compared to normal controls. Post-dialysis these values returned to baseline. (**B**): Genes that remained dysregulated after dialysis treatment. A total of 843 genes were upregulated and 532 genes were downregulated. (**C**): Genes expressed following incubation in normal plasma spiked with indoxyl sulfate compared with their expression levels in cells treated with pre-dialysis and post-dialysis plasma. A total of 908 genes were upregulated and 571 genes were downregulated. (**D**): 81.5% of upregulated genes that were not normalized by dialysis were mimicked by addition of indoxyl sulfate to normal plasma. (**E**): 80.4% of downregulated genes that were not normalized by dialysis were mimicked by addition of indoxyl sulfate to normal plasma.

The expression of 500 genes that were dysregulated (either increased or decreased) when renal tubular cells were incubated with pre-dialysis plasma was “normalized” following incubation in plasma following a standard hemodialysis (Figure 1A). Importantly, the expression of roughly 1500 genes remained dysregulated (Figure 1B), many to a greater degree following hemodialysis, providing evidence that the uremic toxin or toxins were poorly dialyzable. Importantly, roughly 80% of the gene dysregulation observed with uremic plasma was mimicked by the addition of indoxyl sulfate to control plasma at a concentration comparable to that observed in uremic plasma (Figure 1C–E). Not shown here, it was observed that gene dysregulation was blocked when probenecid, an inhibitor of OATs, was added to the incubate, confirming the role of OAT transporters in mediating the effects of indoxyl sulfate.

Among the dysregulated genes noted to be more highly expressed were several recognized as possible mediators of tissue injury [39]. Recognizing that uremic toxicity is almost certainly polygenic, a panel of genes related to the toxicity of Transforming Growth Factor-beta (TGF-beta) was identified in subjects with and without Residual Renal Function undergoing standard hemodialysis. Residual Renal Function (RRF) is considered to represent the secretion of solutes and fluid by proximal tubular OATs [40,41].

**Figure 2:**
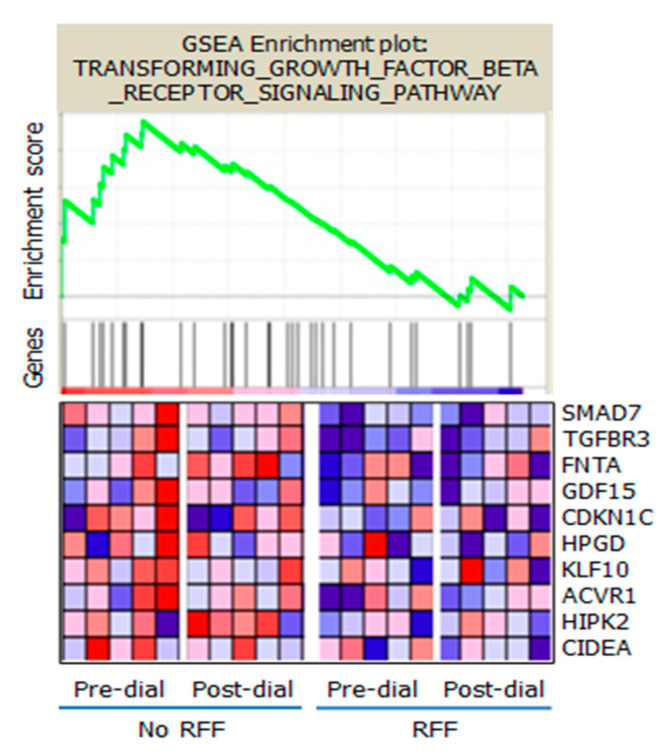
From Jhawar S et al. PLoS ONE 10(3): e0118703.Fig 5. The expression of genes in the TGF receptor pathway identified by Pavlidis Template Matching [27] in the DAVID data base [31] and subjected to Gene Set Enrichment Analysis (GSEA) [32,33]. The 10 uremic patients were dichotomized into those without RRF (*n* = 5) on the left and those with RF (*n* = 5) on the right. The GSEA enrichment score curve (top panel) for TGF-beta receptor signaling (34 genes), indicates 10 genes as core-enriched (mapped on ranked list left of the peak on the enrichment score curve). The activation of these 10 genes was greater following incubation in pre- and post-dialysis (Pre-Dial, Post-Dial) plasma of subjects without RRF than in subjects with RRF (bottom panels, red in heat map = high mRNA abundance, blue = low abundance).

The expression of genes in the TGF receptor pathway was identified by Pavlidis Template Matching. The 10 uremic patients were dichotomized into those without RRF (*n* = 5) on the left and those with RF (*n* = 5) on the right (Figure 2). The GSEA enrichment score curve (top panel) for TGF-beta receptor signaling (34 genes) indicates 10 genes as core-enriched (mapped on the ranked list left of the peak on the enrichment score curve). The activation of these 10 genes was greater following incubation in pre- and post-dialysis (Pre-Dial, Post-Dial) plasma of subjects without RRF than in subjects with RRF (bottom panels, red in heat map = high mRNA abundance, blue = low abundance). It is notable that there was little change in the concentration of these genes with hemodialysis.

## 4. The Evolving Concept of Uremic Toxicity

**Figure 3:**
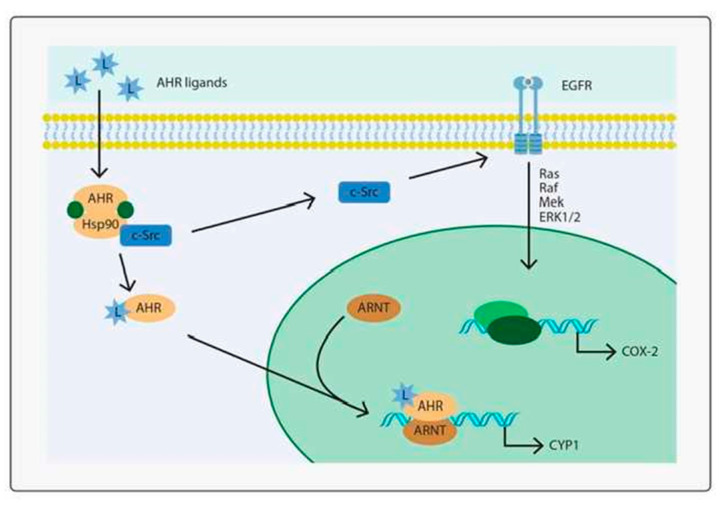
From Vogeley, C. Int. J. Mol. Sci. 2019, 20, 6005. AHR-dependent signaling pathways. In its inactive state, AHR is part of a multiprotein complex consisting of different chaperone molecules and, possibly, tyrosine kinase c-Src. Upon ligand binding, the complex dissociates and AHR translocates into the nucleus, where it dimerizes with ARNT to form a transcriptionally active complex and induces the expression of target genes, for instance encoding CYP1 cytochrome p450 isoforms. In addition to this canonical signaling pathway, the ligand-driven dissociation of the cytosolic AHR multiportion complex stimulates c-Src activity, which is followed by activation of EGFR and downstream MAPK mitogen activated protein kinase signaling, resulting in the transcriptional induction of another set of genes, such as cyclooxygenase-2 (COX-2).

Evidence has accumulated demonstrating that indoxyl sulfate and other protein-bound solutes mediate tissue injury in isolated tissues, providing abundant evidence that indoxyl sulfate and probably other “uremic toxins” are not simply waste products, but that “uremic toxins” probably function as transcription factors [42]. Following entry through OATs, indoxyl sulfate binds to the Aryl Hydrocarbon Receptor (Figure 3) [42,43]. This is followed by the release of heat-shock proteins (Hsp90) and the passive transport of the complex of IS indoxyl sulfate/AHR aryl hydrocarbon receptor across the nuclear membrane to bind with the Aryl Receptor Nuclear Translocator (ARNT). This complex binds to specific xenobiotic response elements, XREs on chromosomes, to activate gene expression. Early studies identified these XREs as responsible for the removal of foreign antigens. Over the past several years, there has been increasing evidence that the genes activated are often responsible for the response to endogenous antigens [43,44] as well.

**Figure 4:**
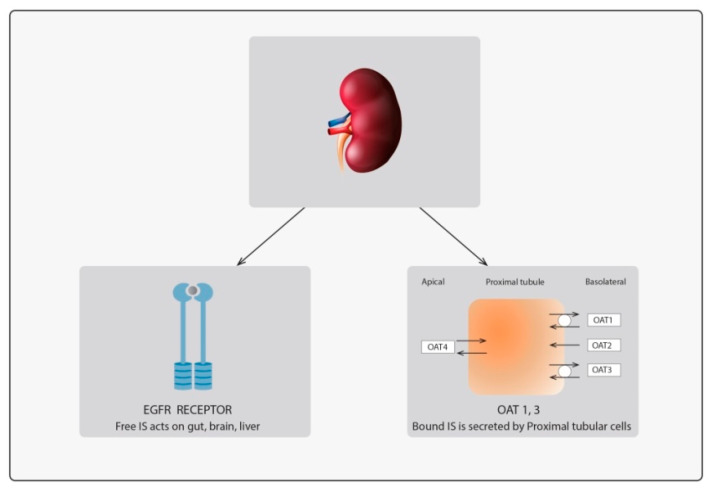
The renal handling of indoxyl sulfate excretion is complex. The free fraction is filtered in the glomeruli, whereas the fraction bound to albumin is (mostly) retained in the blood and thus efferent arterioles. Indoxyl sulfate is actively transported by Organic Anion Transporters (OATs) in the proximal tubuli. There is autoregulation of the tubular handling, as indoxyl sulfate activates Aryl Hydrocarbon Receptors (AhR) and upregulates expression of the organic anion transporters in the proximal tubuli. Non-canonical signaling via the epidermal growth factor receptor (EGFR) plays a role in other tissues such as gut, liver and brain. More recently, we demonstrated a role for EGFR-mediated signaling in proximal tubuli contributing to autoregulation of tubular handling.

Importantly, further studies indicate that OATs are not the sole mechanism responsible for the transport of indoxyl sulfate and similar protein-bound solutes into cells. Recent data indicates that free (unbound) indoxyl sulfate and other aryl hydrocarbons can bind to the Epithelial Growth Factor Receptor (EGFR) of cells lacking OATs (Figure 3) and induce a signaling cascade that results in ARNT translocation and initiate gene transcription [45,46]. These findings suggest that the earlier identification of indoxyl sulfate as a uremic toxin whose plasma concentration was governed by renal tubular OAT transport was incomplete. The fuller view of indoxyl sulfate identifies it as a signaling molecule controlling functions in non-renal tissues. The transport of indoxyl sulfate by renal OATs serves to maintain the concentration of indoxyl sulfate; with nephron loss, the concentration of indoxyl sulfate, both free and bound, increases and effects become evident in non-renal tissues (Figure 4).

While the study of Jhawar et al. [39] demonstrated the dysregulation of a very great number of genes in cultured renal tubule cells by indoxyl sulfate, it is likely that the effect of signaling by free indoxyl sulfate on tissues that do not bear OAT receptors might be more selective. How this “selectivity” might be regulated is not evident but might be considered analogous to the way that the response of tissues, all bearing the same genome, respond to specific signals. These actions can be viewed as “remote sensing and signaling”. The Remote Sensing and Signaling Theory [47,48] is a general theory that attempts to explain how multi-specific, oligo-specific and monospecific transporters and enzymes work together, along with regulatory proteins (e.g., nuclear receptors, kinases), to achieve optimal levels of hundreds if not thousands of small molecules in different body tissues (e.g., CNS central nervous system, kidney, liver, placenta) and body fluids, e.g., blood, CSF cerebrospinal fluid, urine, bile, amniotic fluids.

## 5. Conclusions

Taken together, our current understanding of the biological role of endogenous OAT ligands suggests that the concept of uremic toxicity might be an oversimplification. Our body is well-adapted to low concentrations of the vast majority of these solutes, with dedicated signaling cascades to respond to fluctuations in biological availability. The Remote Sensing and Signaling Theory postulates that while the kidney, through OATs, serves to facilitate the excretion of indoxyl sulfate, free indoxyl sulfate can serve to activate Aryl Hydrocarbon Receptor (AHR) though the Epithelial Growth Factor Receptor (EGFR) and other receptors in distal sites to mediate a host of effects, some beneficial to the organism.

The loss of kidney function disturbs this delicate balance. This implies that some uremic toxins might actually have beneficial effects at low concentrations, as discussed by Vanholder and coworkers in this Special Issue of Toxins. We therefore propose a refined definition to include these insights.

“A uremic toxin is a molecule whose concentration is increased due to the loss of kidney clearance to values above the healthy state, and exerts adverse biological activity at these concentrations”.

The maxim by Paracelsus thus still holds great truth: “All things are poison, and nothing is without poison; the dosage alone makes it so a thing is not a poison” [49]. The consequence of this shift in thinking would suggest that treating uremia should be focused on the restoration of these signaling cascades, rather than maximizing the clearance of toxins. Masereeuw provides insights on how to achieve this goal.

## Data Availability

Not applicable.
